# The Snoopy Sign Behind a MINOCA: Complete Congenital Absence of the Pericardium

**DOI:** 10.3390/diagnostics16193159

**Published:** 2026-09-29

**Authors:** Claudiu-Florin Rășinar, Silvia Ana Luca, Florin Mircea Giru, Simina Crisan, Corina Maria Vasile, Constantin Tudor Luca

**Affiliations:** 1 Department of Cardiology, Institute of Cardiovascular Diseases, 300310 Timișoara, Romania; 2 Doctoral School, “Victor Babeș” University of Medicine and Pharmacy, 300041 Timișoara, Romania; 3 Cardiology Department, “Victor Babeș” University of Medicine and Pharmacy, 300041 Timișoara, Romania; 4 Department of Cardiovascular Surgery, Institute of Cardiovascular Diseases, 300310 Timișoara, Romania; 5 Department of Radiology, Columbia University, New York, NY 10032, USA; 6 Research Center of the Institute of Cardiovascular Diseases, 300310 Timișoara, Romania

**Keywords:** MINOCA, congenital absence of the pericardium, pericardial agenesis, Snoopy sign, multimodality imaging, cardiac magnetic resonance, T2 mapping, extracellular volume, coronary computed tomography angiography

## Abstract

**Background:** Myocardial infarction with non-obstructive coronary arteries (MINOCA) requires further investigation to identify the underlying mechanism. Congenital absence of the pericardium is a rare anomaly, usually asymptomatic and incidentally diagnosed. **Case Presentation:** A 57-year-old woman presented with prolonged resting chest pain, inferior T-wave inversion, and markedly elevated high-sensitivity cardiac troponin I (4393 ng/L). Transthoracic echocardiography was limited by non-diagnostic standard views and revealed marked cardiac displacement with preserved left ventricular systolic function. Coronary angiography showed unobstructed coronary arteries but raised suspicion of an anomalous right coronary origin. Coronary computed tomography angiography excluded this anomaly and demonstrated complete absence of the pericardium with marked leftward cardiac displacement. Cardiac magnetic resonance confirmed the anatomical diagnosis and showed preserved biventricular systolic function, no late gadolinium enhancement, and diffusely elevated T2 and extracellular volume, consistent with acute non-ischemic myocardial injury. The patient recovered with conservative management and remained asymptomatic at follow-up. **Conclusions:** Complete congenital absence of the pericardium may be identified during the evaluation of an acute MINOCA-like presentation. Unusual echocardiographic windows, marked cardiac displacement, and difficult coronary engagement should raise suspicion of this anomaly. Multimodality imaging is essential for defining the anatomy, excluding associated coronary abnormalities, and characterizing myocardial injury.

## 1. Introduction

Myocardial infarction with non-obstructive coronary arteries (MINOCA) is a condition with multiple underlying causes, defined by clinical evidence of myocardial infarction (MI) with normal or mildly obstructed coronary arteries (≤50% stenosis) on angiography. MINOCA accounts for approximately 10% of acute coronary syndromes and may result from several underlying mechanisms [1].

The 2018 Fourth Universal Definition of Myocardial Infarction and subsequent position statements characterize this presentation as MINOCA and define it as a provisional diagnosis rather than a definitive label. Based on the underlying pathological process, causes can be broadly classified as epicardial or microvascular. Epicardial causes include plaque disruption that may not be evident on coronary angiography, coronary artery spasm, and spontaneous coronary artery dissection. Microvascular mechanisms may be further divided into intrinsic causes, such as microvascular spasm, Takotsubo syndrome, and coronary embolism, and extrinsic causes, particularly myocarditis. In intrinsic microvascular disorders, myocardial injury may result from impaired microcirculation due to vasoconstriction and/or lumen obstruction. In myocarditis, by contrast, myocardial edema may compress the microvasculature externally.

The prognosis of patients with MINOCA varies considerably depending on the underlying cause, with certain clinical subgroups carrying a higher risk of adverse outcomes [1,2,3,4,5,6,7].

Congenital pericardial agenesis (CPA) is a rare condition characterized by partial or complete absence of the pericardium, with an estimated prevalence of 0.002–0.004% and a male predominance [8,9,10]. It occurs when the pleuropericardial membranes fail to develop normally during embryogenesis, most often following early regression of the left common cardinal vein (duct of Cuvier) [9,11]. Without pericardial support, the heart moves more freely within the chest, usually resulting in leftward displacement [9,12,13].

Complete absence is generally considered benign, while partial (foraminal) left-sided defects carry a genuine risk of herniation and strangulation of a cardiac chamber, coronary compression, malignant arrhythmia, and sudden death [12,14,15,16,17,18].

Ischemic or infarct-like presentations of congenital pericardial defects have been described predominantly in partial defects, in which the free pericardial rim can entrap the ventricle or compress an epicardial coronary artery [19,20]. Complete absence, which lacks such a rim, is generally regarded as benign and, in the largest systematic review to date, never required surgical correction [21]; its association with an acute presentation fulfilling MINOCA criteria has rarely been reported. We report the case of a female patient who presented with suspected acute coronary syndrome and a marked increase in troponin levels, despite the absence of obstructive coronary artery disease. The subsequent MINOCA work-up revealed complete congenital absence of the pericardium, diagnosed in her sixth decade of life. The initial echocardiogram already showed features suggestive of this diagnosis, although they were initially attributed to technical limitations. Marked cardiac levorotation also made coronary angiography difficult to interpret and raised suspicion of an anomalous coronary origin. Cardiac magnetic resonance imaging confirmed the absence of the pericardium and helped clarify the mechanism behind the acute presentation. The case is reported in accordance with the CARE guideline [22].

## 2. Case Report

### 2.1. Presentation and Initial Assessment

A 57-year-old woman presented to the department four hours after the sudden onset, at rest, of constrictive anterior chest pain radiating to the left shoulder and jaw, accompanied by fatigue and transient light-headedness. The pain was continuous and progressive, neither pleuritic nor postural, with no dyspnea, syncope, or palpitations. A diagnosis of non-ST-elevation myocardial infarction was made, and she was transferred the same night to a tertiary cardiovascular center, arriving still symptomatic.

There was no history of prior cardiac symptoms or previously documented cardiovascular disease. The patient had not undergone thoracic or cardiac surgery, pericardial instrumentation, or significant chest trauma, and reported no fever or viral prodrome in the preceding weeks.

The patient was not receiving any chronic medication, reported no tobacco, alcohol, or illicit drug use, and had no family history of premature cardiovascular disease or sudden cardiac death. Hypertension and dyslipidemia were first identified during the index admission; consequently, age and overweight (height 172 cm, weight 80 kg; body mass index 27.0 kg/m^2^) were the only cardiovascular risk factors recognized at presentation.

On examination, she was afebrile and hemodynamically stable, with a blood pressure of 150/70 mmHg, a regular pulse of 58–63 bpm, and an oxygen saturation of 96–98% on room air. Cardiac auscultation revealed regular heart sounds without murmurs, a third heart sound, or a pericardial friction rub—the latter, in retrospect, consistent with the absence of the pericardium. There was no jugular venous distension or peripheral edema, and lung auscultation was unremarkable.

The admission electrocardiogram showed sinus rhythm at 58 bpm, with T-wave inversion in leads II, III, and aVF, no ST-segment elevation, and a QTc interval of 427 ms. The P wave was negative in lead V1 and biphasic in V2–V3, a pattern retrospectively attributable to an altered atrial depolarization vector secondary to marked cardiac rotation. At discharge, sinus bradycardia (41 bpm) and inferior T-wave inversion persisted, without any rate-limiting medication.

### 2.2. Laboratory Findings

On admission, high-sensitivity cardiac troponin I was 4393 ng/L (upper reference limit 29 ng/L), confirming acute myocardial injury. Creatine kinase, its MB fraction, aspartate aminotransferase, and lactate dehydrogenase were maximal on admission and fell progressively over the next three days, describing the rise-and-fall pattern of an acute myocardial event (Figure 1). On the second day, N-terminal pro-B-type natriuretic peptide was 1724.5 pg/mL. Markers of systemic inflammation were minimal: erythrocyte sedimentation rate was 15 mm/h, and leukocyte counts remained within normal limits (8.67, 8.62, and 6.84 × 10^3^/µL on days 1–3). Full blood count, renal function, electrolytes, and coagulation parameters were unremarkable. The lipid profile revealed total cholesterol of 202 mg/dL, LDL-cholesterol of 129 mg/dL, and triglycerides of 136 mg/dL.

### 2.3. Imaging During the Index Admission

During admission, three diagnostic studies were conducted: chest radiography, transthoracic echocardiography, and invasive coronary angiography. Although each study revealed findings pertinent to the eventual diagnosis, they were initially interpreted as technically challenging or unremarkable.

A portable anteroposterior chest radiograph obtained in the sitting position on admission showed an enlarged cardiac silhouette markedly displaced to the left. The right heart border was barely visible beyond the vertebral spine, and the left cardiac contour extended toward the lateral chest wall. The left ventricular border appeared elongated and flattened, and a radiolucency separated the aortic knob from the pulmonary artery segment (Figure 2). No pulmonary congestion, pleural effusion, or focal consolidation was observed. This combination of findings—levoposition of the heart, an indistinct right heart border, and lung interposed inferiorly, an elongated, flattened left ventricular border, and lung interposed between the aorta and the pulmonary artery—corresponds to the “Snoopy dog” appearance described in congenital absence of the pericardium. At the time of admission, however, these findings were interpreted only as a technically limited portable film in a patient with an atypical cardiac position.

Transthoracic echocardiography, performed on the first day of hospitalization, was technically very challenging. Standard parasternal long- and short-axis views, as well as conventional apical views, were nondiagnostic in the usual left lateral decubitus position because the heart could not be visualized in its expected retrosternal position. Interpretable images were obtained only with the transducer placed markedly laterally, near the posterior axillary line, and image quality remained suboptimal even from this window (Figure 3, Videos S1 and S2).

From the available views, the left ventricle was not dilated, with an end-diastolic diameter of 45 mm, an interventricular septal thickness of 13 mm, and a posterior wall thickness of 12 mm. Left ventricular systolic function was preserved (planimetric ejection fraction approximately 50%), with no regional wall motion abnormalities. Mild degenerative mitral regurgitation and mild functional tricuspid regurgitation were noted. There was no evidence of aortic valve disease (peak aortic velocity, 1.2 m/s) or pulmonary hypertension. Mild left ventricular diastolic dysfunction was present (mitral E-wave velocity, 0.45 m/s; A-wave velocity, 0.72 m/s). The ascending aorta measured 35 mm, and no pericardial effusion was identified.

In retrospect, the inability to obtain standard echocardiographic views reflected the abnormal cardiac position rather than a technical limitation.

Given the clinical presentation, electrocardiographic changes, and elevated cardiac biomarkers, the patient was managed as a non-ST-elevation acute coronary syndrome in accordance with the 2023 ESC guidelines [5] and underwent invasive coronary angiography via right radial access. The coronary circulation was right-dominant, with no angiographically significant stenoses involving the left main coronary artery, left anterior descending artery, left circumflex artery, right coronary artery, or their major branches. Selective engagement of the right coronary artery was unusually difficult using standard Judkins catheters and was ultimately achieved with a 6F internal mammary catheter in a 30° right anterior oblique/6° caudal projection. Based on the technical difficulty encountered during catheterization and its apparent origin on angiography, the right coronary artery was initially considered to arise from the left coronary sinus. No culprit lesion, intracoronary thrombus, coronary dissection, or spontaneous coronary spasm was identified. In the absence of obstructive coronary artery disease, we therefore classified the case as MINOCA (Figure 4; Video S3).

### 2.4. Cross-Sectional Imaging After Discharge

As the absence of obstructive coronary artery disease left the mechanism of myocardial injury unclear, and an anomalous coronary origin had been suspected, ECG-gated coronary computed tomography angiography was performed after discharge using a 384-slice dual-source scanner, at a mean heart rate of 43 bpm. Both coronary arteries arose normally from their respective sinuses of Valsalva, excluding the initially suspected anomalous origin of the right coronary artery. The Agatston calcium score was 0, and no stenotic lesions were identified in the left main coronary artery, left anterior descending artery, first or second diagonal branches, left circumflex artery, obtuse marginal branch, right coronary artery, posterior descending artery, or posterolateral branches.

The key finding was extracardiac. No pericardial tissue could be identified at any level. The heart was markedly displaced and rotated to the left, with lung tissue interposed between the inferior cardiac border and the left hemidiaphragm, corresponding to the “Snoopy” configuration seen on the chest radiograph. There was no pleural effusion, and there was no mediastinal, hilar, or axillary lymphadenopathy. The trachea and main bronchi were patent. The ascending aorta, aortic arch, and descending aorta were normal in caliber, without aneurysmal dilatation, stenosis, or dissection. The brachiocephalic trunk, subclavian arteries, and common carotid arteries were also patent. Incidental findings included an azygos lobe fissure, thoracic spondylosis, and bilateral hypodense thyroid nodules measuring up to 15 mm, for which endocrinological evaluation was recommended.

Cardiac magnetic resonance (CMR) imaging was performed 16 days after symptom onset and one week after computed tomography, using native and contrast-enhanced cardiac and thoracic sequences. Biventricular systolic function was preserved, with no global or regional wall motion abnormalities and no intracavitary masses or thrombi (Figure 5, Video S4). However, both ventricles were clearly enlarged, without increased left ventricular myocardial mass. Atrial volumes were within normal limits, valvular regurgitation was mild, and no intracardiac shunt was detected (Qp/Qs, 1.02). Quantitative CMR findings are summarized in Figure 6.

Tissue characterization provided additional insight. Late gadolinium enhancement was absent throughout the ventricular myocardium, with no evidence of a subendocardial or transmural ischemic scar or of the subepicardial and mid-wall patterns typically associated with myocarditis. In contrast, parametric mapping showed globally increased T2 values (59.9 ± 4.3 ms) and an elevated extracellular volume fraction (31%; hematocrit, 37.2%). Consistent with the CT findings, no pericardial layer could be identified, and the heart was again markedly rotated and displaced to the left, producing the characteristic “Snoopy sign” (Figure 5, Video S4). A thin layer of left posterior pleural fluid was noted, while the remaining lung parenchyma and mediastinum were unremarkable.

### 2.5. Diagnosis, Management and Follow-Up

Based on the clinical presentation, marked troponin elevation, absence of obstructive coronary artery disease on angiography, and cross-sectional imaging, the final diagnosis was acute myocardial injury presenting as MINOCA due to congenital complete absence of the pericardium. Cardiac magnetic resonance imaging revealed diffuse myocardial edema and interstitial expansion, with no evidence of infarction scar. The diagnostic pathway is summarized in Figure 7.

During hospitalization, the patient received guideline-directed therapy for presumed NSTE-ACS, including dual antiplatelet therapy, low-molecular-weight heparin, an angiotensin-converting enzyme inhibitor, a high-intensity statin, and gastric protection. Beta-blockers were withheld because of resting sinus bradycardia. Chest pain resolved completely, no arrhythmias were recorded on continuous telemetry, and the patient was discharged in stable condition on the fourth hospital day, with a blood pressure of 110/70 mmHg, a heart rate of 55 bpm, and an oxygen saturation of 98% on room air.

At outpatient follow-up, the patient remained asymptomatic, with no recurrence of chest pain, no exercise limitation, and no further troponin elevation. Once the diagnosis had been established and an atherothrombotic mechanism was considered unlikely, the antithrombotic regimen was de-escalated. Management of cardiovascular risk factors, including blood pressure control and lipid-lowering therapy, was continued.

Pericardial surgery was not indicated, and a conservative strategy was adopted, with regular clinical follow-up and repeat imaging reserved for new or progressive symptoms. The incidentally detected thyroid nodules were referred for endocrinological evaluation.

### 2.6. Preparation of Figures

Figure 2 is the admission radiograph exported from a DICOM viewer (RadiAnt) at full display resolution. Figure 3, Figure 4 and Figure 5 consist of single frames extracted without resampling from the cine loops provided as Videos S1–S4; the frames used are identified in the figure legends. Processing was limited to cropping, masking patient identifiers (examination date and time, operator initials, and study identifiers, which were also masked in the Supplementary Videos), arranging images into multi-panel layouts, placing panel letters over masked, non-identifying viewer text, and adding numeric labels and arrows. We drew annotations as geometric overlays at positions specified by the authors who acquired and reported each study; we made no pixel-level modifications to the image content (e.g., denoising, sharpening, interpolation, inpainting, or generative synthesis), and we applied no adjustments beyond standard display windowing. Figure 5 was enlarged threefold by integer pixel replication (nearest-neighbor) for legibility, without interpolation. A pixel-wise comparison with the source images confirmed that all image pixels were unchanged except those covered by annotations or identifier masks. Two authors compared each final figure side by side with the original study to confirm that the anatomical content was unchanged. Figure 1, Figure 6 and Figure 7 are graphical representations of the authors’ clinical data; the role of generative artificial intelligence in figure preparation is detailed in the Acknowledgments.

## 3. Discussion

The diagnostic work-up in this case illustrates the reasoning underpinning current guidelines, which emphasize that MINOCA should not be regarded as a definitive diagnosis. All three formal criteria were fulfilled: the universal criteria for acute myocardial infarction, the absence of coronary stenosis ≥ 50%, and the lack of an alternative clinically apparent cause at the time of angiography [2,3,4]. Had the diagnostic evaluation ended at this stage, the patient would have been labeled as having an unexplained myocardial infarction and prescribed 12 months of dual antiplatelet therapy, while the congenital abnormality and diffuse myocardial edema would have remained undetected. Registry data indicate that MINOCA carries a clinically meaningful risk of adverse outcomes, largely determined by the underlying etiology. Accordingly, elucidating the underlying mechanism is essential for guiding appropriate management, not a purely academic exercise [6,7].

Cardiac magnetic resonance is the pivotal investigation in the work-up of MINOCA, identifying the underlying cause in approximately three-quarters of patients and discriminating between myocardial infarction, myocarditis, takotsubo syndrome, and other non-ischemic cardiomyopathies [4,7,23]. In the present case, CMR provided detailed myocardial tissue characterization and confirmed the anatomical abnormality that had compromised the echocardiographic assessment since admission.

Congenital pericardial defects result from premature involution of the left common cardinal vein, reducing blood supply to the developing pleuropericardial membrane and preventing complete closure. These defects range from small foraminal openings to complete bilateral absence of the pericardial sac (Table 1).

The reported prevalence in autopsy series is below 1 in 10,000 (0.002–0.004%) [8,9,10]; however, the true prevalence remains uncertain, as most affected individuals are asymptomatic. Left-sided defects are most common, whereas right-sided defects account for about one-sixth of cases and complete bilateral absence for about one-tenth. Associated congenital anomalies, including atrial septal defect, patent ductus arteriosus, bicuspid aortic valve, bronchogenic cyst, pulmonary sequestration, and diaphragmatic hernia, are present in a substantial minority of patients [12,14,15,16,17,24].

**Table 1:** Anatomical variants of congenital pericardial defects and their clinical implications. Reported frequencies vary considerably across series because most cases are asymptomatic and diagnosis depends on the imaging modality used.

Anatomical Variant	Reported Frequency	Clinical and Imaging Features	Usual Management
Complete left-sided absence	Most common variant [9,12,17]	Levoposition, hypermobile heart, paradoxical septal motion; usually benign [12,18,25,26]	Conservative; reassurance, activity generally unrestricted [14,15,16]
Partial (foraminal) left-sided defect	Uncommon, but clinically the most dangerous [14,17]	Herniation and strangulation of the left atrial appendage, left atrium or left ventricle; coronary compression; sudden death [14,16,17,27]	Pericardioplasty, defect enlargement or appendectomy if symptomatic or herniation documented [14,16,17]
Right-sided defects	≈17% of defects [9,12]	Rarely reported; may be associated with pulmonary or diaphragmatic anomalies [9,12]	Individualized; usually conservative [14,15]
Complete bilateral (total) absence	≈9% of defects [9,12]	Extreme cardiac mobility; positional symptoms; no risk of strangulation [12,22,26]	Conservative [14,15]

Clinically, the main distinction is between complete and partial absence of the pericardium. Complete absence eliminates the normal pericardial anchoring of the heart but does not create a rigid defect margin capable of entrapping a cardiac chamber. As a result, this condition is generally benign and managed conservatively. In contrast, partial left-sided defects present a greater risk because the free edge of the residual pericardium may strangulate the left atrial appendage, left atrium, or left ventricle, compress a coronary artery, and lead to myocardial ischemia, malignant arrhythmias, or sudden cardiac death. Patients with partial defects may require pericardioplasty, surgical enlargement of the defect, or appendectomy [14,15,16,17].

For our patient, neither computed tomography nor cardiac magnetic resonance imaging identified any residual pericardial tissue, supporting classification as a complete and prognostically favorable absence. However, imaging may not definitively exclude a vestigial right-sided pericardial remnant. Differentiating between complete left-sided absence and complete bilateral absence may not be possible without surgical or post-mortem confirmation.

Positioned against the existing literature, the present case adds incremental information in four respects. First, previously reported ischemic or infarct-like presentations have almost invariably involved partial or hemi-pericardial defects, in which myocardial injury was attributed to ventricular herniation and strangulation or to compression of an epicardial coronary artery by the rigid pericardial rim, frequently requiring surgery [19,20]; in our patient, the defect was complete, so this mechanism was anatomically excluded. Second, although chest pain is the most frequent symptom of congenital pericardial absence [21], it is rarely accompanied by a troponin rise fulfilling formal MINOCA criteria, as in this case (151 times the upper reference limit). Third, CMR provided complete tissue characterization, including T2 and extracellular volume mapping, which has seldom been documented in complete pericardial absence [12,25]. Fourth, cardiac malposition produced a false impression of an anomalous right coronary origin on invasive angiography, subsequently refuted by coronary CT angiography. Together, these features show how an unrecognized congenital substrate can confound each step of the standard MINOCA work-up.

Multiple imaging modalities contributed to the diagnosis in this case, with the initial findings preceding clinical suspicion of congenital absence of the pericardium. On chest radiography, characteristic features include marked leftward displacement of the cardiac silhouette, loss of the right heart border, prominence of the pulmonary artery segment, and interposition of lung tissue between the inferior cardiac border and the left hemidiaphragm, producing the classic “Snoopy dog” appearance [25].

Echocardiographic findings may be suggestive but are easily misinterpreted as technical limitations related to poor acoustic windows. Standard parasternal and apical views may be difficult or impossible to obtain, often requiring more lateral or posterior transducer positioning. Additional features include displacement of the apparent cardiac apex, excessive cardiac mobility within the thorax, abnormal or paradoxical interventricular septal motion, and apparent right ventricular enlargement [12,18,28]. Changes in cardiac position and echocardiographic windows with postural variation—from the left lateral decubitus to the supine or right lateral position—may provide an additional diagnostic clue [12].

Cross-sectional imaging is essential for diagnosis and for defining the extent of the defect. Computed tomography and cardiac magnetic resonance imaging can demonstrate absence of the expected pericardial line, interposition of lung tissue between the aorta and pulmonary artery or between the heart and diaphragm, and abnormal leftward displacement and rotation of the heart [15,28,29]. In our patient, CT and CMR demonstrated concordant features strongly supporting complete congenital absence of the pericardium.

Difficulties in obtaining conventional echocardiographic windows—particularly when accompanied by marked cardiac displacement or mobility—should not automatically be attributed to poor acoustic access. Recognition of this pattern may provide an early clue to congenital pericardial absence and should prompt cross-sectional imaging for anatomical confirmation.

Cardiac magnetic resonance imaging illustrated that both ventricles had volumes well above the normal reference range. Despite this, biventricular ejection fraction remained preserved, and left ventricular mass and atrial volumes were normal. There was no significant valvular regurgitation, no intracardiac shunt (Qp/Qs 1.02), and no clinical evidence of heart failure. This pattern is well recognized in cases of congenital absence of the pericardium and is best explained by the loss of normal pericardial constraint on diastolic filling rather than by true cardiomyopathic remodeling.

The intact pericardium is essential for limiting ventricular distensibility and maintaining diastolic ventricular interdependence. In its absence, ventricular volumes, particularly those of the right ventricle, may increase. Septal motion can become abnormal, and the heart may swing and rotate within the mediastinum [18,24,26,28]. This is clinically relevant because similar volumetric findings in a patient with an intact pericardium would prompt evaluation for dilated cardiomyopathy. Additionally, right ventricular findings in partial pericardial defects have been shown to closely mimic arrhythmogenic right ventricular cardiomyopathy, sometimes meeting established Task Force diagnostic criteria [24].

Invasive coronary angiography was misleading in this case. Selective cannulation of the right coronary artery proved challenging, requiring an internal mammary catheter and an unconventional angiographic projection. The right coronary artery was initially interpreted as arising from the left coronary sinus, a finding with potentially important ischemic and prognostic implications that would normally prompt further evaluation and, in selected cases, consideration of surgical management [30,31]. Subsequent coronary computed tomography angiography, however, confirmed normal coronary ostial anatomy.

This can be explained by the marked leftward displacement and rotation of the heart associated with complete pericardial absence. The altered cardiac orientation changes the spatial relationship between the aortic root and standard fluoroscopic views, potentially making both catheter behavior and the apparent coronary ostial location misleading. Thus, in the presence of marked cardiac malposition, an apparent anomalous coronary origin on invasive angiography should be interpreted with caution and confirmed by cross-sectional imaging before further management decisions are made.

Conversely, unexpected difficulty in selective coronary engagement, particularly when non-standard catheters or projections are required, may itself provide a clue to abnormal cardiac orientation.

The underlying mechanism of troponin elevation remains unclear, as CMR imaging did not reveal a definitive etiology. The absence of late gadolinium enhancement (LGE) suggests that myocardial infarction with irreversible injury and typical focal myocarditis are unlikely. Nevertheless, both T2 mapping and extracellular volume (ECV) measurements were diffusely elevated. The updated 2018 Lake Louise Criteria indicate that abnormal T2- and T1-based markers support myocardial inflammation [32,33]. These findings suggest diffuse non-ischemic myocardial injury rather than acute myocardial infarction. The clinical presentation may therefore be classified as a MINOCA mimic, as outlined in the Fourth Universal Definition of Myocardial Infarction and the American Heart Association scientific statement [2,4]. Additionally, the complete absence of the pericardium precludes a pericardial component of an inflammatory syndrome; no pericardial rub or effusion was observed.

Interpretation of these findings requires caution. CMR was conducted 16 days after symptom onset, a period during which edema associated with acute resolving myocardial injury may have diminished. Furthermore, diffuse elevations in mapping parameters are less specific than regional abnormalities. T2 and ECV values are influenced by acquisition technique, scanner vendor, field strength, and local reference ranges. Mapping was acquired during marked sinus bradycardia (41 bpm), a condition known to influence T1 estimation with commonly used mapping sequences and, consequently, the derived extracellular volume.

A transient ischemic mechanism cannot be definitively excluded. Without the pericardium, excessive cardiac mobility may cause intermittent mechanical stress or traction on the great vessels and coronary arteries, the primary points of cardiac fixation. Chest pain associated with congenital pericardial defects has been linked to several mechanical factors, including traction on pleuropericardial adhesions, loss of normal pericardial support, and excessive torsion or stretching of the great vessels and coronary arteries. Reports have documented persistent chest pain and coronary compression [24,34]. Positional kinking or torsion of an epicardial coronary artery, particularly the right coronary artery, which follows the displaced atrioventricular groove, could also transiently reduce flow without leaving an angiographic or CT correlate, as both studies were acquired supine and at rest. By contrast, a supply–demand mismatch (type 2 myocardial infarction) appears unlikely: the patient was bradycardic and normoxemic, with a normal full blood count and no tachyarrhythmia on telemetry, although the elevated blood pressure at presentation and the enlarged ventricular volumes may have modestly increased myocardial oxygen demand.

Coronary vasospasm, one of the most frequent mechanisms of MINOCA, requires specific consideration [3,4,35]. Intracoronary provocative testing with acetylcholine or ergonovine was not performed, as these tests are not available at our institution, and referral to another centre was not pursued because the patient remained asymptomatic. Epicardial or microvascular spasm therefore cannot be excluded, as diagnosis requires documentation of inducible spasm [36], which is a limitation of the present report. Several observations nevertheless argue against spasm as the sole explanation. No spontaneous spasm was observed during angiography; the patient had no history of rest angina, and chest pain did not recur during follow-up. Moreover, ischemic injury resulting from spasm severe enough to produce a troponin rise of this magnitude would be expected to follow a coronary territory, whereas CMR showed diffuse rather than regional T2 and extracellular volume elevation, with no regional wall motion abnormality and no LGE. Conversely, the inferior T-wave inversion would be compatible with transient right coronary artery ischemia, and spasm triggered by mechanical stress on a hypermobile, unsupported heart remains a plausible contributing mechanism. In comparable presentations, provocative testing, which has been shown to be safe in patients with MINOCA, would help distinguish between these mechanisms and, where unavailable locally, referral to a center performing it should be considered [4,35].

Overall, the findings support a diagnosis of acute myocardial injury with a predominantly non-ischemic CMR pattern in the context of complete congenital absence of the pericardium. Complete symptom resolution, declining cardiac enzymes toward the reference range with no further troponin elevation at follow-up, and the absence of LGE indicative of infarction are consistent with a self-limited myocardial injury without detectable residual scar.

In adults aged 60 years or older, complete congenital absence of the pericardium without complications does not require surgical intervention. Recent expert reviews advocate for conservative management and patient reassurance in such cases [14,15,16,27]. Partial pericardial defects generally require closer clinical surveillance than complete pericardial agenesis because of their higher risk of complications. Surgery may be considered in symptomatic patients, particularly those with persistent or severe chest pain, or when complications occur. Surgical options include pericardioplasty to close the defect, defect enlargement, and resection of the left atrial appendage. Long-term prognosis is not well defined, because the condition is rare and its clinical presentation varies widely [37].

Management should focus on control of modifiable cardiovascular risk factors, such as hypertension and dyslipidemia, and on limiting antithrombotic therapy. This approach aligns with the concept that antithrombotic medication in MINOCA should target the underlying cause rather than be administered routinely [4,7].

Persistent sinus bradycardia is clinically relevant because it limits beta-blocker use. Sinus node dysfunction has been linked to congenital pericardial abnormalities; however, in this case, bradycardia was asymptomatic and did not require particular intervention. Long-term follow-up for this population typically consists of periodic clinical assessments, with repeat imaging reserved for new or progressive symptoms.

## 4. Conclusions

A particular case of acute coronary syndrome with unobstructed coronary arteries required a systematic diagnostic approach, ultimately revealing a congenital anomaly that had remained asymptomatic for fifty-seven years.

This case has three clinical implications. First, myocardial infarction with non-obstructive coronary arteries (MINOCA) should be considered a provisional diagnosis that necessitates comprehensive evaluation to move from a descriptive label to a targeted management strategy. Second, failure to obtain standard echocardiographic windows may itself be a diagnostic clue. When this limitation coincides with a leftward-displaced cardiac silhouette, perform cross-sectional imaging rather than attributing the finding to suboptimal acoustic access. Third, severe cardiac malposition can distort invasive coronary angiography and potentially simulate an anomalous coronary origin; such findings require confirmation with computed tomography before management decisions are revised.

An echocardiogram that cannot be obtained in standard views should not be dismissed as technically limited; it may be the first sign of an absent pericardium.

## Figures and Tables

**Figure 1:**
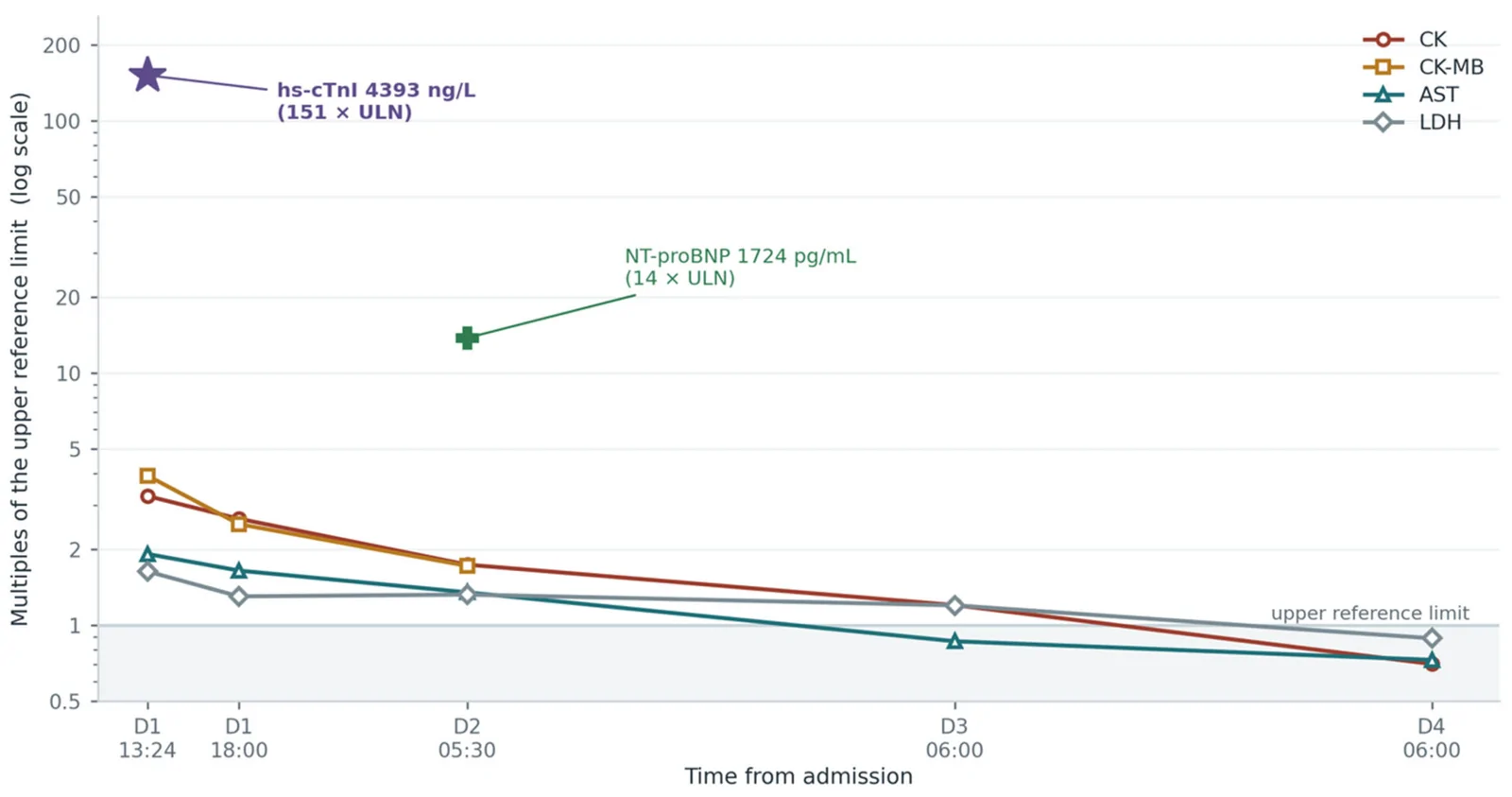
Time course of circulating markers of myocardial injury during the index admission, expressed as multiples of the upper reference limit on a logarithmic scale. Creatine kinase, its MB fraction, aspartate aminotransferase, and lactate dehydrogenase peaked on admission and returned to or toward the reference range by the fourth hospital day. High-sensitivity cardiac troponin I, assayed once on admission, was 151 times the upper reference limit. Shading marks the reference range. hs-cTnI—high-sensitivity cardiac troponin I; CK—creatine kinase; CK-MB—creatine kinase MB fraction; AST—aspartate aminotransferase; LDH—lactate dehydrogenase; NT-proBNP—N-terminal pro-B-type natriuretic peptide; ULN—upper reference limit.

**Figure 2:**
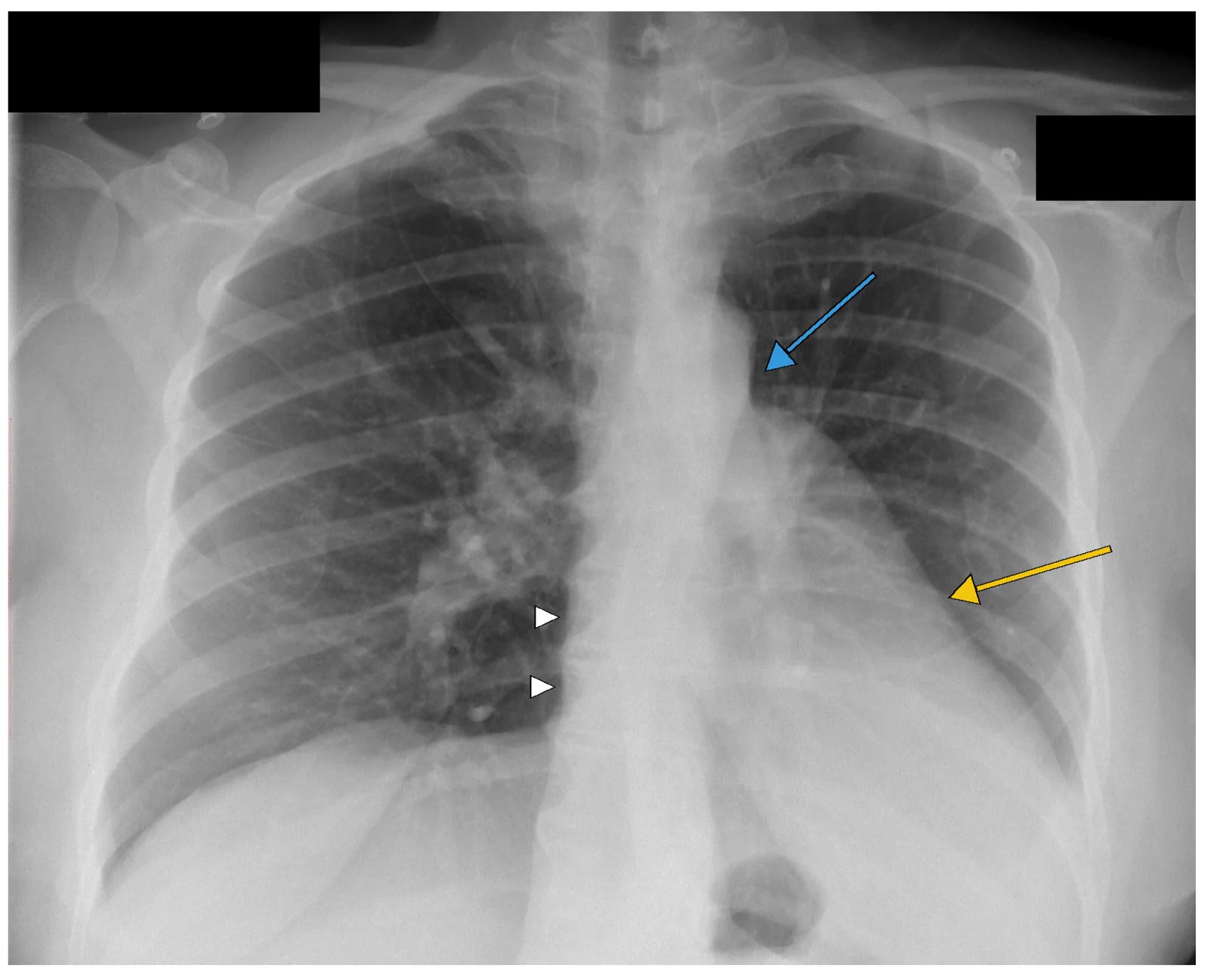
Admission anteroposterior chest radiograph. The cardiac silhouette is displaced into the left hemithorax, with an elongated, flattened left ventricular contour extending toward the lateral chest wall (yellow arrow); the right heart border barely projects beyond the vertebral column (arrowheads); and aerated lung is interposed between the aortic knob and the main pulmonary artery (blue arrow)—together forming the “Snoopy dog” configuration.

**Figure 3:**
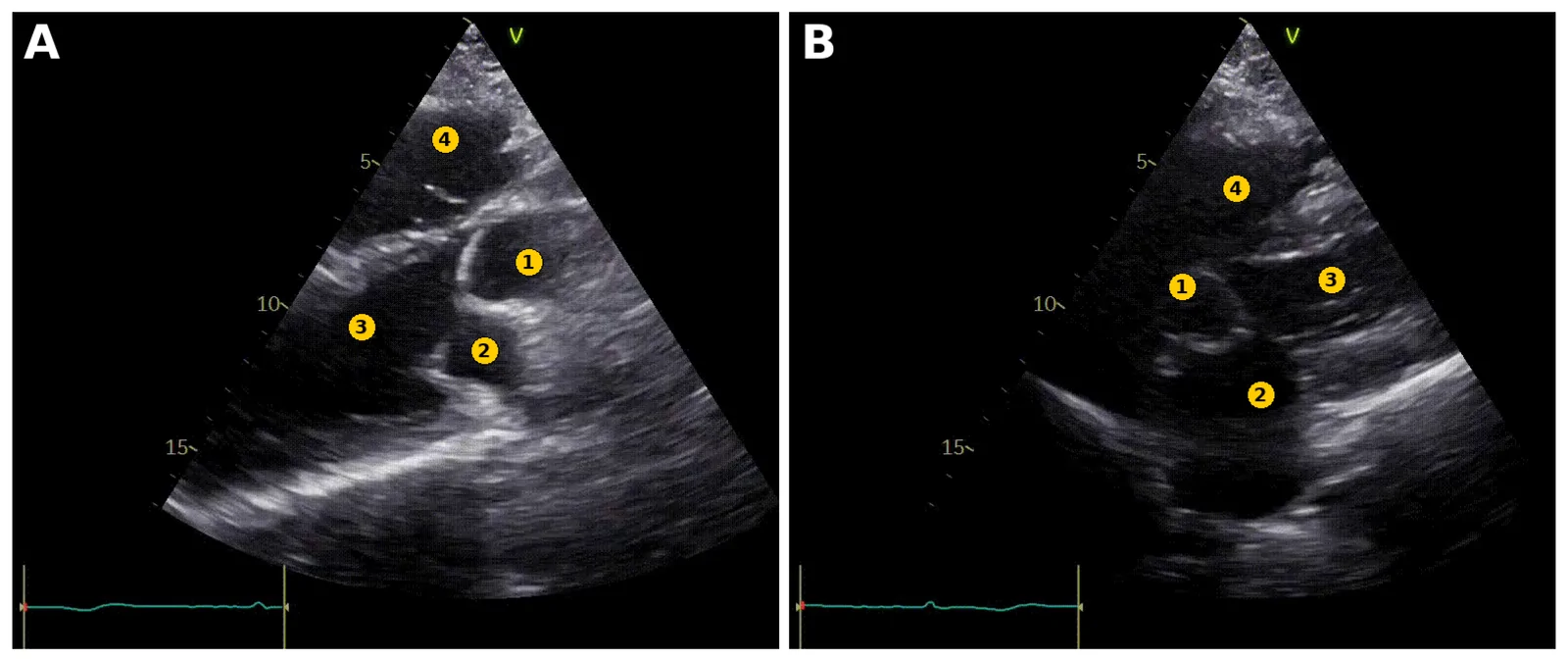
Transthoracic echocardiography acquired during the index admission from a markedly lateral (posterior-axillary) transducer position, the only window from which interpretable images could be obtained. (**A**) Attempted parasternal long-axis equivalent and (**B**) attempted apical four-chamber equivalent. In both cases, conventional parasternal and apical geometries cannot be reproduced, and the chambers appear in highly atypical tomographic planes, reflecting the extreme leftward rotation and displacement of the heart. No pericardial effusion is present. In both panels, numbers indicate the structures visualized in these atypical planes: 1, aortic root; 2, left atrium; 3, left ventricle; 4, right ventricle. The complete cine loops are provided in Videos S1 and S2.

**Figure 4:**
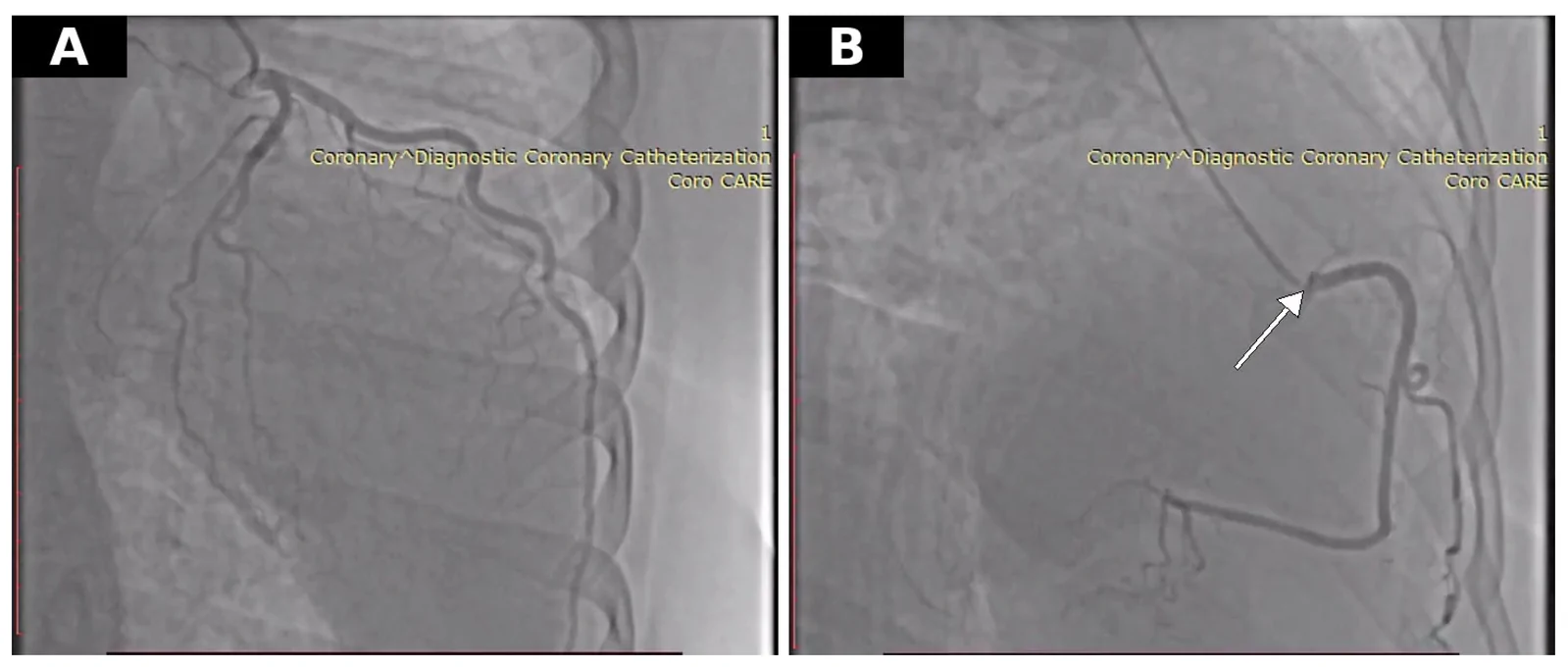
Invasive coronary angiography performed via right radial access. (**A**) Left coronary system and (**B**) right coronary artery, both free of angiographically significant stenoses in a right-dominant circulation. The arrow in (**B**) indicates the right coronary artery ostium, engaged with a 6F internal mammary catheter. The complete cine loops are provided in Video S3. Selective engagement of the right coronary artery required a 6F internal mammary catheter and an atypical projection, and the vessel was initially reported as arising from the left coronary sinus—an impression subsequently refuted by computed tomography (see Section 2.4).

**Figure 5:**
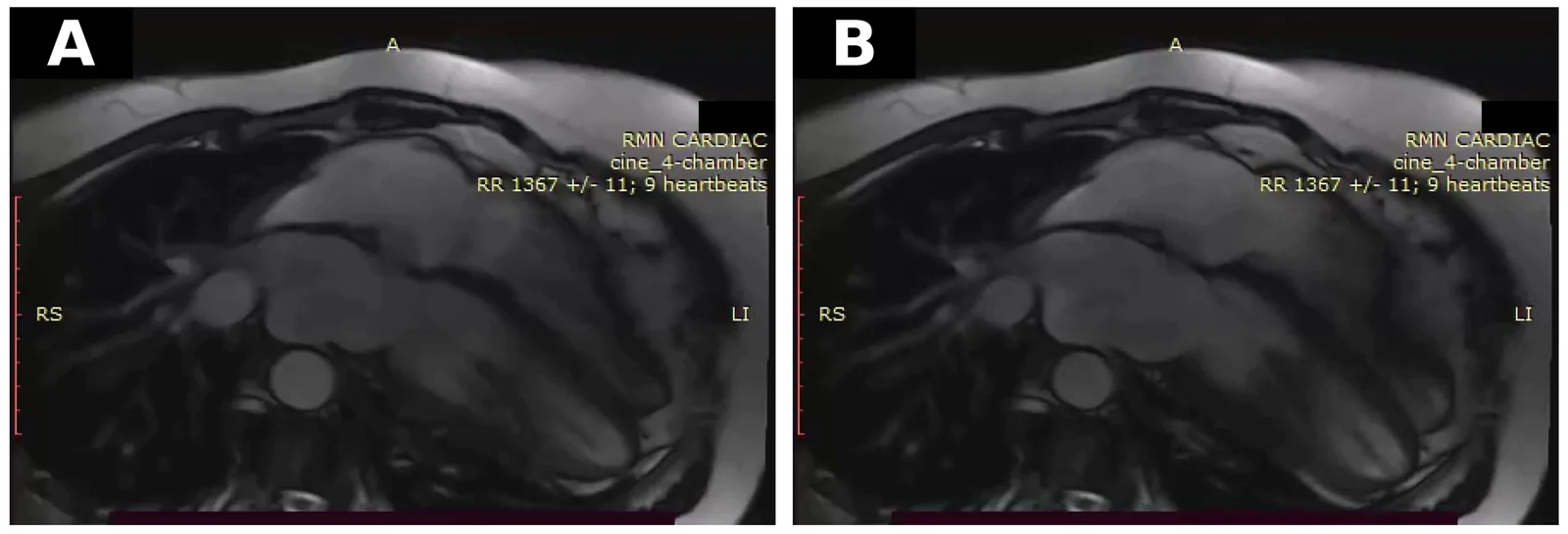
Cardiac magnetic resonance, balanced steady-state free-precession cine imaging in the four-chamber orientation at (**A**) end-diastole and (**B**) end-systole. The heart is markedly rotated and displaced into the left hemithorax and lies against the diaphragm; no pericardial layer can be identified at any level, and both ventricles are enlarged with preserved systolic function. The complete cine loop is provided in Video S4. Orientation labels are those of the scanner display: A, anterior; RS, right–superior; LI, left–inferior.

**Figure 6:**
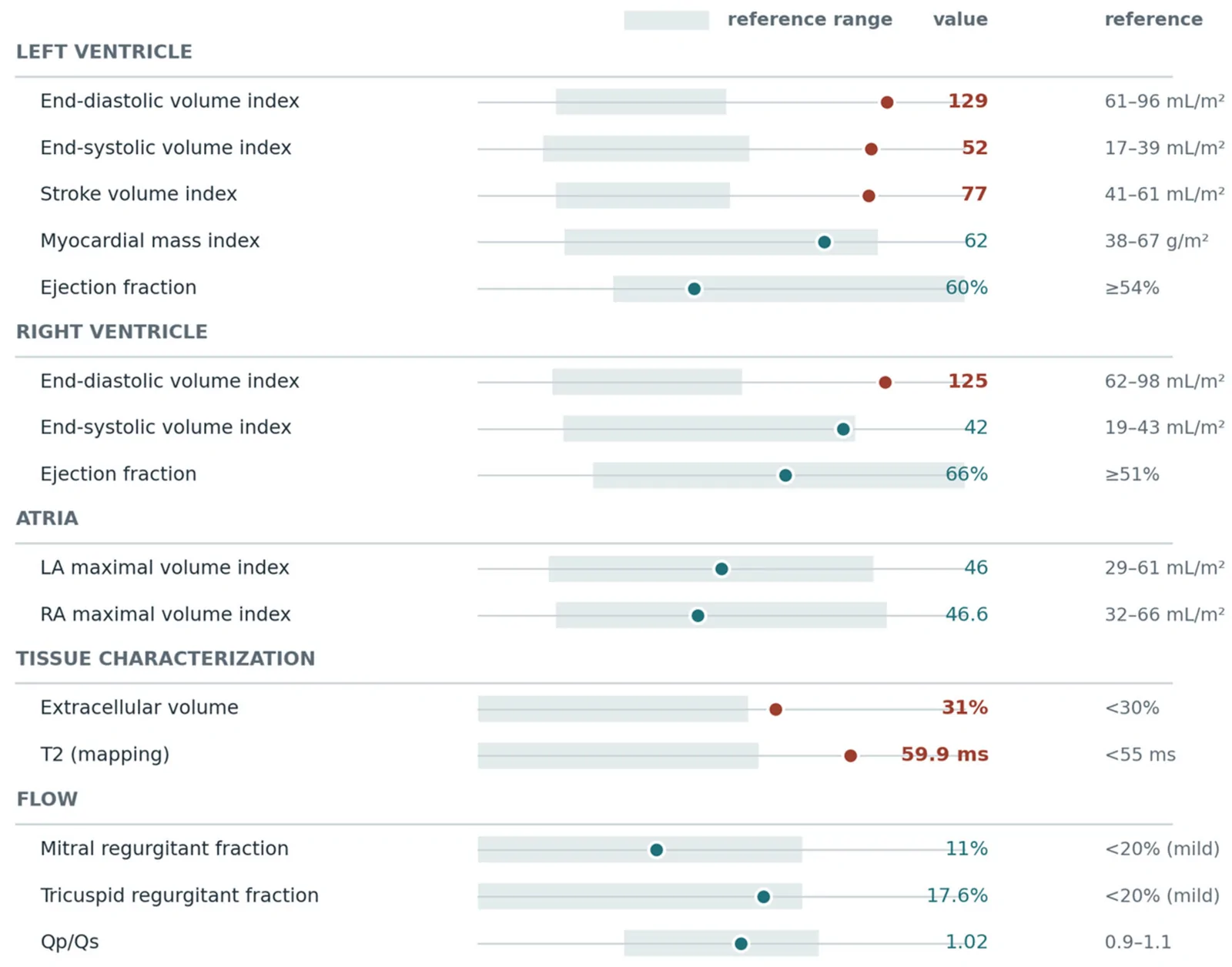
Cardiac magnetic resonance quantitative data. Reference values are indicative sex-specific adult ranges and mapping cut-offs are those of the reporting laboratory; parametric mapping values are sequence-, vendor- and field-strength-dependent and must be interpreted against local normal ranges. LA—left atrium; RA—right atrium.

**Figure 7:**
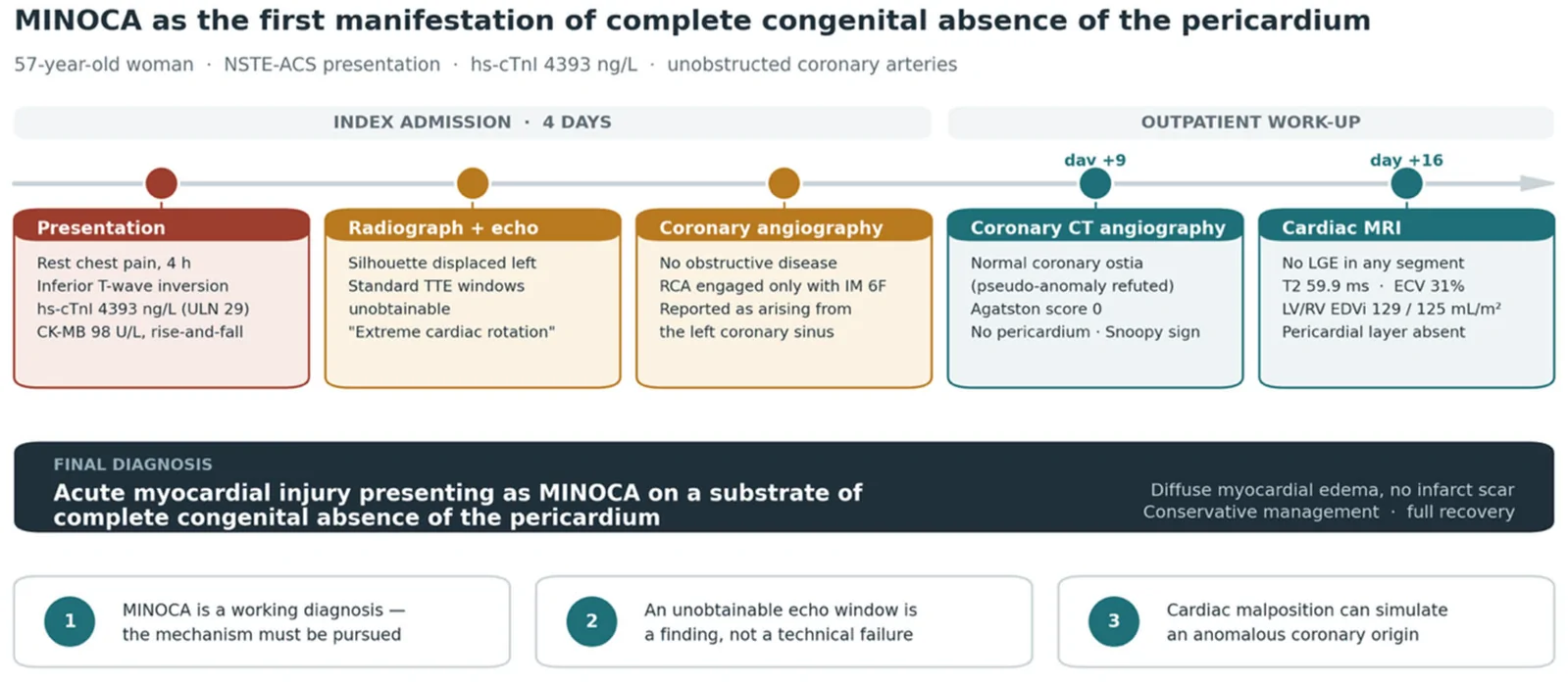
Diagnostic pathway and integration of findings. The index admission identified acute myocardial injury with unobstructed coronary arteries. Subsequent cross-sectional imaging excluded the suspected anomalous right coronary origin, confirmed complete congenital absence of the pericardium, and characterized myocardial involvement. Studies are presented in the order in which they informed the diagnosis. hs-cTnI, high-sensitivity cardiac troponin I; IM, internal mammary; LGE, late gadolinium enhancement; ECV, extracellular volume; EDVi, end-diastolic volume index.

## Data Availability

The data supporting the findings of this case report are contained within the article. Further anonymized clinical and imaging data are available from the corresponding author upon reasonable request, subject to institutional data protection regulations and patient confidentiality.

## Supplementary Materials

The following supporting information can be downloaded at: https://www.mdpi.com/article/10.3390/diagnostics16193159/s1. Videos S1 and S2: Transthoracic echocardiography cine loops acquired from a markedly lateral (posterior-axillary) window: (A) attempted parasternal long-axis equivalent and (B) attempted apical four-chamber equivalent; Video S3: Invasive coronary angiography cine loops of the left coronary system and of the right coronary artery, the latter selectively engaged with a 6F internal mammary catheter; Video S4: Cardiac magnetic resonance cine imaging, four-chamber view, showing marked leftward displacement and rotation of the heart, which lies against the diaphragm and the left lateral chest wall.

## Abbreviations

The following abbreviations are used in this manuscript:

ACS	acute coronary syndrome
AST	aspartate aminotransferase
CK	creatine kinase
CK-MB	creatine kinase MB fraction
CMR	cardiac magnetic resonance
CT	computed tomography
ECG	electrocardiography
ECV	extracellular volume
EDVi	end-diastolic volume index
hs-cTnI	high-sensitivity cardiac troponin I
LA	left atrium
LDH	lactate dehydrogenase
LDL	low-density lipoprotein
LGE	late gadolinium enhancement
LV	left ventricle
MINOCA	myocardial infarction with non-obstructive coronary arteries
NSTE-ACS	non-ST-elevation acute coronary syndrome
NT-proBNP	N-terminal pro-B-type natriuretic peptide
Qp/Qs	pulmonary-to-systemic flow ratio
RA	right atrium
RV	right ventricle
TTE	transthoracic echocardiography
ULN	upper reference limit
